# The role of pH on the biological struvite production in digested sludge dewatering liquors

**DOI:** 10.1038/s41598-018-25431-7

**Published:** 2018-05-08

**Authors:** Francisco Simoes, Peter Vale, Tom Stephenson, Ana Soares

**Affiliations:** 1 Cranfield Water Science Institute, Cranfield University, Cranfield, MK43 0AL UK; 2 Severn Trent Water Limited, Severn Trent Centre, 2 St John’s Street, Coventry, CV1 2LZ UK

## Abstract

Struvite production mediated by bacteria has opened up a new route for phosphorus recovery from wastewater streams but its application to digested sludge dewatering liquors is not yet well understood. This study investigates the growth and biological struvite production of selected bacteria in wastewater liquors with pHs between 5.7 to 9.1. The bacterial growth was assessed through flow cytometry. *Bacillus pumilus, Halobacterium salinarum* and *Brevibacterium antiquum* remained viable at pHs between 5.7 to 9.1 but *B. antiquum* was able to grow at pHs between 7.3 to 7.8. Further analysis allowed the identification of crystals as struvite in tests between pH 7.3 to 8.3. All strains were capable of producing struvite at a range of pHs, but the highest production of 135–198 mg/L was observed for pHs between 7.3 to 8.3. At pHs > 8.3, precipitation of struvite and calcium compounds was observed in inoculated and non-inoculated tests. This study demonstrates that biological struvite production can occur at a wide range of pHs, hence significantly different from chemical struvite precipitation that occurs at pH > 8.3, making it a potentially viable process for phosphorus recovery as struvite from wastewater streams and sludge liquors without strict pH control.

## Introduction

Phosphorus (P) is an essential element in all organisms and a key nutrient in agriculture limiting food production. However, in contrast, P can also act as a pollutant causing eutrophication if released into the natural environments in excess^1^. Wastewater treatment plants (WWTPs) are control points for P discharge to the environment where P is removed from the wastewater by chemical and/or biological processes. Nevertheless, because P is a limiting nutrient, there is growing interest in processes and technologies that allow P recovery from wastewater streams^2,3^.

Phosphorus recovery from sludge dewatering liquors lines has been implemented in several WWTPs through chemically induced struvite precipitation^4^. Sludge dewatering liquors are a by-product of processes aimed at concentrating solids in the sludge. As a result, these liquors only represent a relatively small volume (usually 1–2% of the wastewater flow) but in turn are a nutrient rich stream with high ammonium (99 to 1020 mg N/L) and phosphate (1 to 167 mg P/L)^5–8^. Consequently when the liquors are returned to the head of the WWTP, there is an increase on the P load to the secondary treatment that can be as high as 40% resulting in a constant cycling of nutrients^5^. Chemically induced struvite precipitation in the liquors line has the advantage of reducing the return of P to the head of the works, as well as decreasing operational costs by minimising scale formation (calcium phosphate and struvite) in the pipelines and pumps. Furthermore, this process allows for the recovery of P as struvite which can be used as an agricultural fertiliser^9^. Nevertheless, chemical struvite precipitation requires chemicals to increase the pH to values above 8.5, and to correct the amount of available magnesium to molar concentrations above or equal to the available phosphate^4^. The increase in operational costs due to chemical consumption can sometimes result in difficulties justifying the implementation of a sidestream reactor for chemical struvite production. Taking advantage of the potential for high pH variability of sludge liquors and to minimise costs associated with the addition of chemicals, pH increase through CO_2_ stripping through increased agitation or aeration is also used in full scale processes for liquors from biological nutrient removal (BNR) sites^6^.

As an alternative process to chemical struvite precipitation, a recent study demonstrated the ability of selected bacteria to form bio-struvite in activated sludge liquors and in dewatering liquors, opening a completely new route for P recovery via biomineralisation^10^. Biomineralisation is a widespread phenomenon in nature. Examples of bio-minerals range from the bones and teeth in vertebrates, to the silicate shells in diatom algae and the crystalline magnetite in magnetotactic bacteria^11^. The most widespread bio-minerals are calcium phosphate and calcium carbonate, but others such as struvite, iron oxides, and silicates have been reported, as well as crystalline magnetite produced by magnetotactic bacteria^12^. The most common function of bio-minerals is to provide structural support in the form of skeletons, protection in the form of shells and to provide storage and a source of nutrients. Lowenstam & Weiner (1989)^13^ defined two main mechanisms for biomineralisation, depending on the level of control exerted by microorganism: biologically induced mineralisation (BIM), and biologically controlled mineralisation (BCM). In BIM, the precipitation of minerals is a collateral consequence of the reaction between extracellular ions and metabolic products extruded across, or into the cell wall. The mineral products of BIM are expected to have a heterogeneous range of crystal and chemical properties, and may be found closely associated with the cell wall. The biomineralisation mechanisms of the struvite forming bacteria are still poorly understood and process development and application to wastewater has not yet been explored in detail.

Several microorganisms have been investigated for the ability to form biological struvite (bio-struvite), amongst other bio-minerals using synthetic media^14^. Precipitation of bio-struvite was shown to be frequent in strains capable of producing calcite precipitates^15^. No relationship was found between the taxonomic status of a given strain and its ability to form bio-struvite^16^ as this property has been reported in aerobic, chemoorganothrophic, halotolerant and halophilic bacterial strains^14^.

In WWTPs, the occurrence of struvite within aerobic granular sludge has been reported as being induced by alginate-like exopolysaccharides isolated from extra polymeric substances collected from the granules^17^. Recently, the cultivation of *Bacillus pumilus* and *Brevibacterium antiquum* on sludge dewatering liquors showed promising results, producing bio-struvite crystals reaching 250 μm in size within 10 days^10^. Previously reported advantages of biological struvite recovery in wastewater sludge dewatering liquors include: struvite recovery from wastewaters with low PO_4_-P/L content (7.5, and 30.0 mg PO_4_-P/L in settled wastewater and sludge dewatering liquors), low phosphorus content in the effluent (<2.1 mg PO_4_-P/L), no need to add chemicals, large struvite crystals are produced, facilitating product recovery from wastewater (>250 μm)^10^. Regarding the microorganisms investigated in this study, the biomineralisation mechanisms in synthetic solutions were BCM for *Brevibacterium antiquum* whilst *Bacillus pumilus* and *Halobacterium salinarum* have been reported to follow a BIM mechanism^18^. *B. pumilus* and *B. antiquum* are aerobic gram-positive bacteria whilst *H. salinarum* is a facultative anaerobe *Archaea*. Both *B. antiquum and H. salinarum* are halotolerant microorganism that are able to proliferate in environments containing high sodium chloride (NaCl)^18^. The selected microbial strains have been reported to grow at pHs between 5–9 and mesophile temperature range from 22–34 °C^17^.

This work aims at investigating the impact of pH variability in digested sludge dewatering liquors on the ability of selected bacteria, *B. pumilus*, *H. salinarum*, and *B. antiquum*, to grow and produce bio-struvite towards bringing bio-struvite production through biomineralisation to WWTPs.

## Materials and Methods

### Source of microorganisms and sludge dewatering liquors

Three pure microbial strains were purchased from commercial culture collections: *Bacillus pumilus* (GB 43, LGC Standards, Middlesex, UK), *Halobacterium salinarum* (DSM 671, German Resource Centre for Biological Material, Brunswick, Germany), and *Brevibacterium antiquum* (DSM 21545, German Resource Centre for Biological Material, Brunswick, Germany).

Sludge dewatering liquors were collected from a full-scale WWTP with a 500,000 population equivalent, with biological nutrient removal (BNR) applied as a secondary treatment. Primary and secondary sludge produced onsite and imported from nearby municipal WWTP (40% v/v) were stabilised in anaerobic digesters. After digestion, the sludge was stored in a holding tank, from 10 to 27 days, before dewatering. A horizontal centrifuge decanter was used to dewater the sludge from typical values of 7% solids to 22% solids content. Cationic polymer, anti-scaling and antifoaming agents were used to aid the centrifugation process. The sludge dewatering liquors were collected at the liquid line from the centrifuge.

### Microorganisms cultivation

Starter cultures of the selected bacteria were produced for the experiments completed in sludge dewatering liquors. The selected bacteria were inoculated at a ratio of 10% (v/v) in B41 synthetic media (4 g/L of yeast extract, 2 g/L of magnesium sulphate heptahydrate, and 2 g/L of di-potassium hydrogen phosphate), incubated in conical flasks, at room temperature (20–22 °C), under agitation at 150 rpm (Stuart SSL1, Fisher Scientific, Loughborough, UK) for 4 days.

In order to measure the bacterial growth and isolate the effect of the selected bacteria towards the bio-struvite production, the sludge dewatering liquors were sterilised. Two different sterilisation techniques were used: autoclave (121 °C, 20 min), and filter sterilisation method (disposable 0.45 μm vacuum filtration set-up, Nalgene, Fisher Scientific, Loughborough, UK); to assess the impact of different sterilisation techniques on the pH of the dewatering liquors.

### Growth of selected bacteria in sludge dewatering liquors

Autoclaved digested sludge dewatering liquors were inoculated with selected bacteria from starter cultures and incubated in conical flasks, at room temperature (20–22 °C), under agitation at 150 rpm (Stuart SSL1, Fisher Scientific, Loughborough, UK). Samples of 10 mL were collected immediately after inoculation and after 2, 4, 5, 6, 7, and 10 days for ammonium (NH_4_), phosphate (PO_4_), magnesium (Mg^2+^), and pH analysis. At the end of the incubation, the solid precipitates produced were collected by filtration, dried and identified. All cultures were carried out in duplicate. Control tests with autoclaved sludge dewatering liquors and no added bacteria were also monitored.

### Effect of pH in the bacterial growth and precipitate production

To assess the optimal pH to grow the selected bacteria and produce precipitates such as struvite, the sludge dewatering liquors were buffered at 5 different pH values using 3 different buffers reported to not affect bacterial growth^19^: N-(morpholino)-ethane-sulfonic acid (MES), N-2-hydroxy ethylpiperazine-N9-propane-sulfonic acid (EPPS), and cyclohexylamino-ethane-sulfonic acid (CHES) (Fisher Scientific, Loughborough, UK). Buffers were added at a 10% (v/v) ratio to 54 mL of magnesium-supplemented sludge dewatering liquors autoclaved in capped serum bottles. The quantities and concentrations of the chemicals used to control the pH of the sludge dewatering liquors are summarised in Table 1.

**Table 1:** Buffer and acid or base additions to control the pH of sludge dewatering liquors.

Buffer	Concentration (mM)	pH correction	pH
MES	50	1200 µL of 2 M HCl	5.7
EPPS	10	180 µL of 2 M HCl	7.3
EPPS	10	60 µL of 2 M NaOH	7.8
EPPS	10	300 µL of 2 M NaOH	8.3
CHES	50	1200 µL of 2 M NaOH	9.1

MES, N-(morpholino)-ethane-sulfonic acid; EPPS, N-2-hydroxy ethylpiperazine-N9-propane-sulfonic acid; CHES, cyclohexylamino-ethane-sulfonic acid.

For the inoculum, bacterial cells were centrifuged from 5 mL of the starter cultures (Sanyo MSE Falcon 6/300 centrifuge, 2400 g, 5 min) and resuspended in 5 mL sterile 0.9% NaCl solution. The tests were completed in triplicates in sacrificial serum bottles under the same conditions described above.

Bacterial growth was assessed by flow cytometry using a live/dead cells staining method that provides the number of cells with intact membranes^20^. Intact cells counts were measured using a SYBR Green I and propidium iodide dye mixture with an incubation period of 10 min at 37 °C^21^. Measurements were taken using a BD Accuri C6^®^ flow cytometer equipped with a 488 nm solid-state laser (Becton Dickinson U.K. Ltd., Oxford, UK). When necessary, samples were diluted with filter sterilised Evian mineral water (0.1 μm) (Evian, Évian-les-Bains, France). Specific fixed regions of the density plots of green fluorescence (533 nm), and red fluorescence (>670 nm) were used for distinction between the stained intact microbial cells and instrument noise or sample background, as described by Gatza *et al*.^20^. Data were processed using the BD Accuri C6^®^ software.

### Bio-struvite production rate

To follow the formation of bio-struvite over 10 days of incubation period, sacrificial bottles were prepared by autoclaving 45 mL of the sludge dewatering liquors in 120 mL glass serum bottles. The pH of the sludge dewatering liquors was controlled by two different processes: half of the bottles were autoclaved with a cotton-plug to keep sterile conditions and pH corrected using sulphuric and hydrochloric acids, at 2 N concentration to control the pH to values between 7.9 and 8.2 (Experiment A); the other half of the bottles were kept closed with rubber stoppers during autoclaving and incubation to limit degassing of CO_2_ allowing for stable pH values between 7.8 and 8.2 (Experiment B).

For the inoculum, bacterial cells were centrifuged from 5 mL of the starter cultures (Sanyo MSE Falcon 6/300 centrifuge, 2400 g, 5 min) and resuspended in 5 mL sterile 0.9% NaCl solution, in order to avoid the addition of PO_4_, NH_4_ and Mg^2+^ present in the B41 media.

Each sacrificial bottle was tested for pH, PO_4_, NH_4_, Mg^2+^ and the precipitates formed were collected by filtration, dried and identified. In Experiment B, each bottle was also tested for total phosphorus (TP) and dissolved oxygen. To maximise the potential for struvite production, the sludge dewatering liquors were supplemented with magnesium, making phosphate the limiting ion for struvite formation. Magnesium sulphate was added to a final concentration of 60.0 mg/L as Mg^2+^.

### Precipitates isolation, quantification and identification

After incubation, the media was vacuum-filtered through a previously dried (2 hours, 37 °C) and weighted 10 µm aperture nylon mesh sheet (Plastok Associates, Birkenhead, UK). The solid precipitates recovered were washed with a small quantity of washing water (deionised water adjusted to pH 10 with 1 M NaOH) and allowed to dry at 37 °C for 2 hours prior to being weighed. The precipitates were identified using high resolution scanning electron microscopy (SEM) with energy dispersive X-ray spectroscopy (EDX) (scanning electron microscope XL 30 SFEG, Phillips, The Netherlands).

### Analytical methods

The concentrations of NH_4_, PO_4_, TP, and chemical oxygen demand (COD), were measured using Merck cell test kits according to the manufacturer instructions. Mg^2+^ was analysed using an atomic absorption spectrophotometer (Analyst 800, Perkin Elmer Ltd, Beaconsfield, UK) equipped with an air/acetylene burner system. The pH was measured with a Fisherbrand hydrous 300 pH meter (Fisher Scientific, Loughborough, UK) immediately after sampling. Values presented details of the mean and standard deviation of duplicate or triplicate tests.

## Results and Discussion

The sludge dewatering liquors used in this study were analysed for typical wastewater quality parameters (Table 2). Nutrients were measured at concentrations of 825 ± 66 mg NH_4_-N/L, 44.5 ± 2 mg PO_4_-P/L, and 15.2 mg Mg^2+^/L. The characteristics of the studied sludge dewatering liquors were similar to others originating from biological nutrient removal sites^8,22^ and thus, it can be considered standard.

**Table 2:** Characteristics of the sludge dewatering liquors collected from a full scale WWTP (average of 10 samples).

pH	COD(mg/L)	NH_4_(mg N/L)	PO_4_ (mg P/L)	Mg^2+^ (mg/L)
7.8	455 ± 3	825 ± 66	44.5 ± 2	15.2

**Figure 1:**
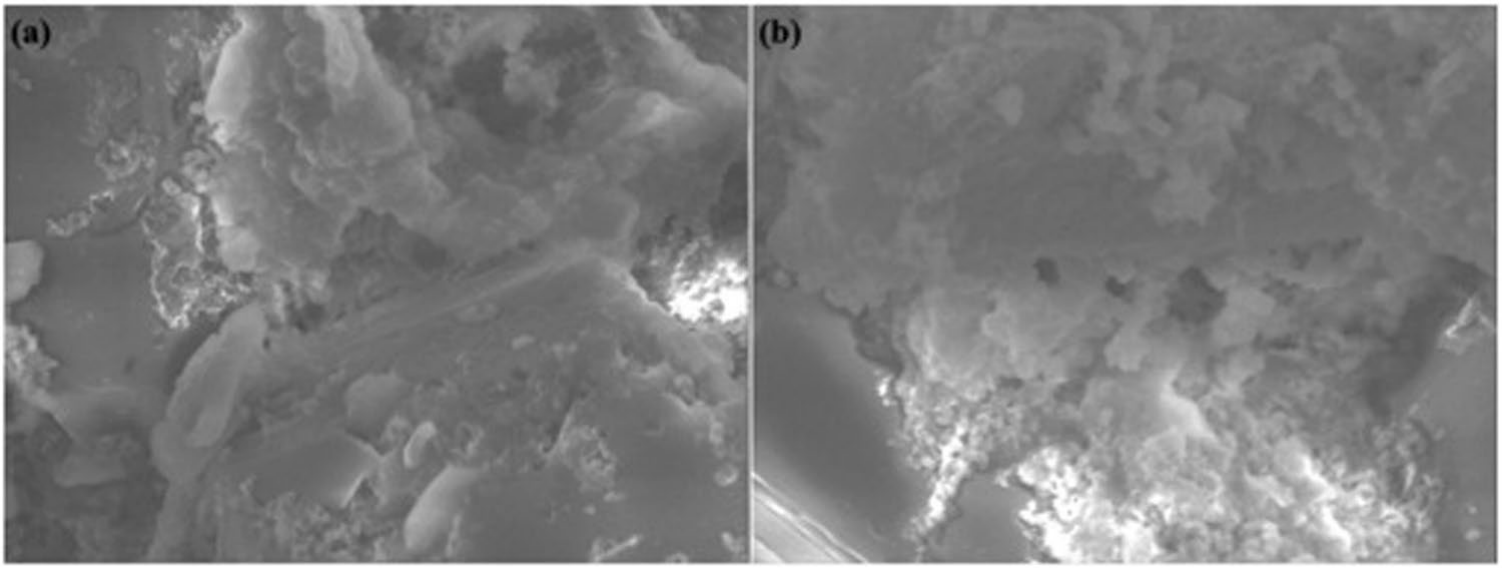
Electron microscope images of the solid precipitate collected at the end of the incubation period of sludge dewatering liquors without pH control at 2000× magnification: (**a**) *B. antiquum*; (**b**) non-inoculated control.

In order to assess the production of bio-struvite in the sludge dewatering liquors, the first test completed was to grow the selected bacteria in the autoclaved sludge dewatering liquors, as previously described by Soares *et al*.^10^. Nevertheless, after 10 days of incubation, no significant differences were found between inoculated and non-inoculated controls, as no bacterial growth was observed and similar precipitate formation was observed in all the bottles. A detailed investigation indicated that the pH in the sludge dewatering liquors had increased from 7.8 to 9.8 due to CO_2_ degassing (took place through mild aeration as well as various sterilisation methods - see supplemental information). The solid precipitate collected after incubation were similar both in the control and inoculated tests, and did not display crystalline forms typical of struvite (Fig. 1). Given the amorphous nature of the solids collected and the pH of the sludge dewatering liquors measured at 9.4 ± 0.1, the precipitates formed were amorphous calcium compounds, identified by morphological analysis^23^. Liquors from biological nutrient removal sites have been shown to have a high propensity to form scaling in the pipes as a result of high concentrations of phosphate together with pH variations caused by degassing of CO_2_ after anaerobic digestion in turbulent flow regions^6,24^.

The degassing of CO_2_ from sludge dewatering liquors has been described previously as a suitable method to increase the pH of dewatering liquors, but this is not applicable to all WWTPs^5,8^. The sludge dewatering liquors used in this study were obtained from a BNR WWTP where the digested sludge was kept in a holding tank for a long period (10–27 days). Assessing previous studies and the results obtained, it is clear that the source of sludge and the type of post anaerobic digestion treatment can influence dissolved CO_2_ concentrations and consequently impact on the pH sludge dewatering liquors. The pH values of the sludge dewatering liquors were found to be extremely high compared with other values reported in the UK (7.2–7.9)^25^. The pH profile of the sludge dewatering liquors tested in this study was considered atypical. Therefore, sludge storage and pH is identified as a relevant factor that requires careful consideration prior to implementation of P recovery processes such as chemical or biologically induced struvite production.

### Impact of pH in the bacterial growth and bio-struvite production

**Figure 2:**
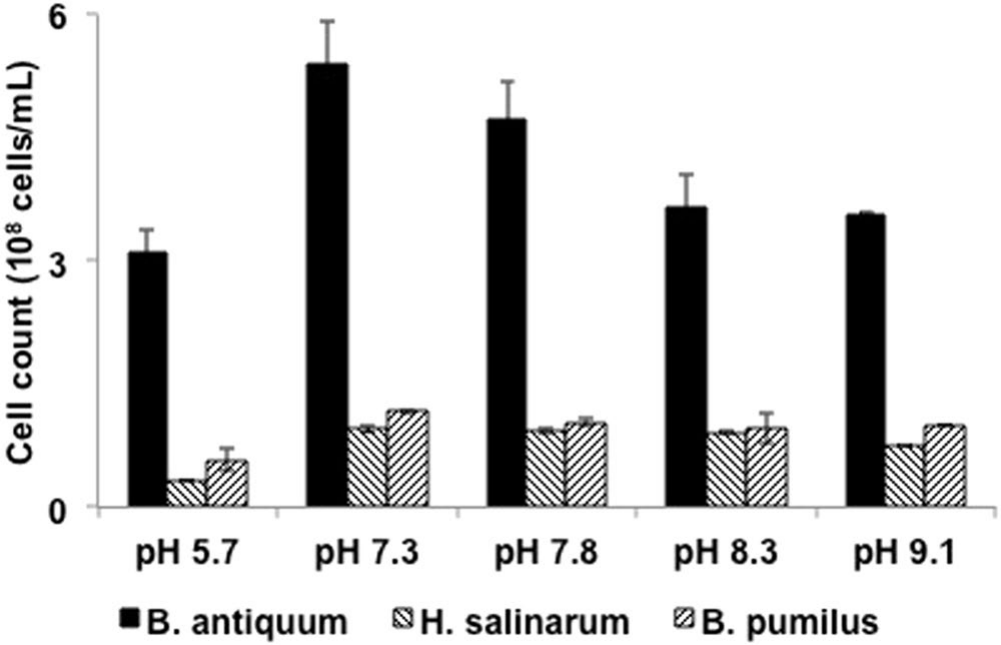
Flow cytometry live cell counts after incubation of the selected bacteria at different pH values.

In order to understand the impact of pH on the growth of selected bacteria and the ability to produce minerals such as struvite, the pH of the sludge dewatering liquors was corrected to different target values: 5.7, 7.3, 7.8, 8.3, and 9.1 using buffers. The results obtained indicate that all bacteria were able to remain viable in sludge dewatering liquors at pH values of 5.7 to 9.1 (Fig. 2). However, the final intact cell counts for *H. salinarum* and *B. pumilus* after 7 days of incubation (9.6 × 10^7^ ± 4.6 × 10^6^ cells/mL for *H. salinarum* and 1.2 × 10^8^ ± 2.5 × 10^6^ cells/mL *B. pumilus* at pH 7.3) were slightly below the initial count (1.2 × 10^8^ ± 3.1 × 10^6^ cells/mL for *H. salinarum* and 2.0 × 10^8^ ± 8.6 × 10^6^ for *B. pumilus*). This suggests that sludge dewatering liquors limited the growth of these bacteria at the studied pH values. Nevertheless the bacterial cell counts remained at the initial values at pHs tested between 7.3–9.1. For *B. antiquum* the initial cell count was 4.7 × 10^8^ ± 7.5 × 10^7^ cells/mL and increased to 5.4 × 10^8^ cells/mL at pH 7.3. and 4.8 × 10^8^ cells/mL at pH 7.8 (Fig. 2). As such, from the three selected bacteria, *B. antiquum* seems to be better adjusted to grow in sludge dewatering liquors and the optimal pH for growth was 7.3–7.8. Published studies on *B. antiquum* have reported growth in synthetic media at pH values between 6.0 and 7.0^26,27^ and between 5 and 9^28^. These published values are in good agreement with the results described in this study and suggest that pH is not a major limiting factor for *B. antiquum* growth in sludge dewatering liquors. From the three investigated bacteria, *B. pumilus* was expected to have the widest pH interval for growth as 16 isolates from a coastal environment were reported to be able to grow at pH values between and 5 and 11^29^. For *H. salinarum*, growth has been reported at pH between 6.0 and 7.0 in synthetic media^27,30^ but optimum growth was reported at pH 7.0^31^.

**Figure 3:**
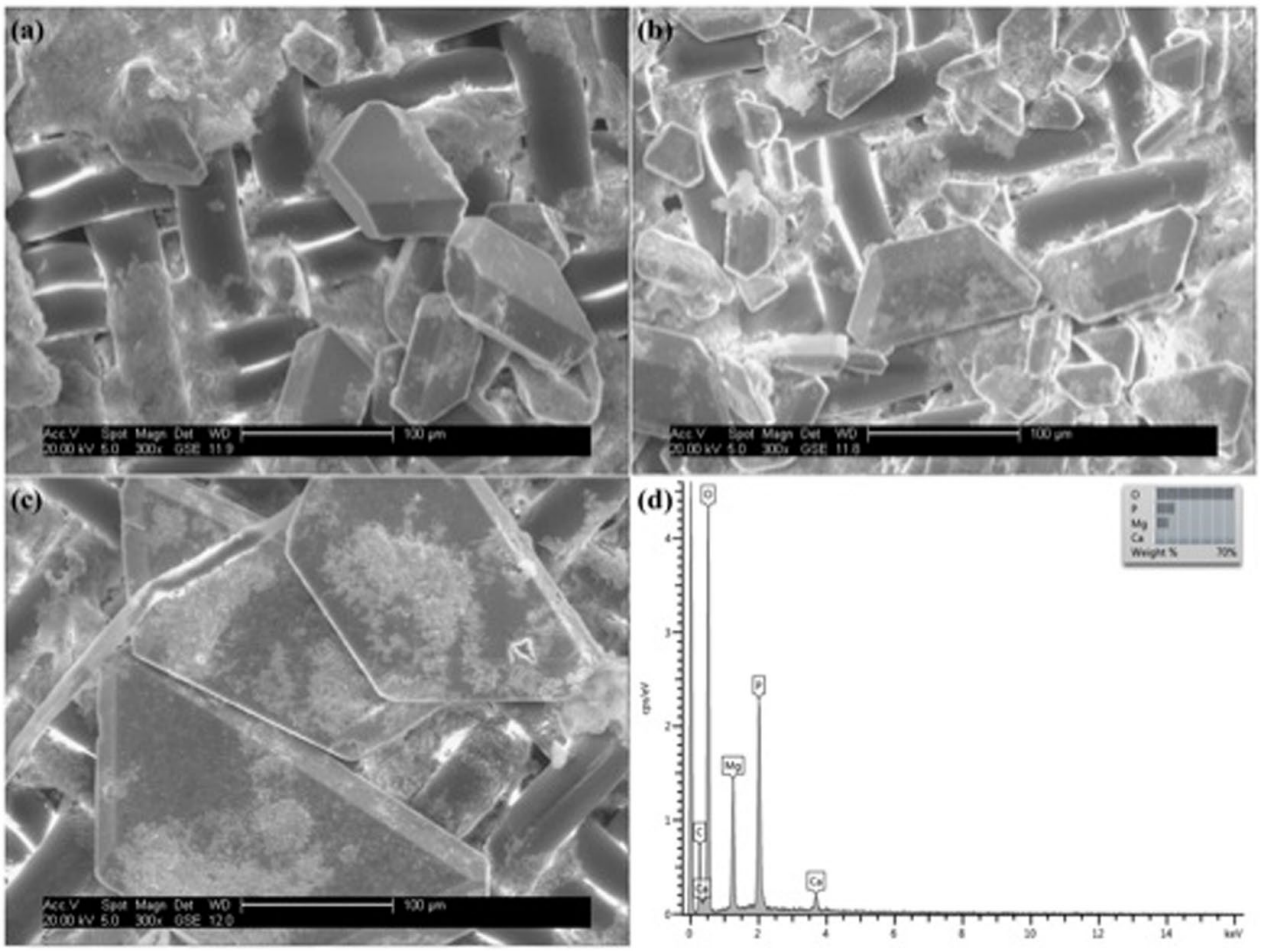
Electron scanning microscope images of the solid precipitate collected at the end of the 10 days of incubation for *B. pumilus* (**a**), *H. salinarum* (**b**), and *B. antiquum* (**c**) at pH 7.3 in sludge dewatering liquors at 300× magnification, and (**d**) example of an energy dispersive x-ray (EDX) microanalysis of the crystals for *B. antiquum* (**c**) with weight percentage of the elemental components of the analysed crystal that allowed the identification of the crystal as bio-struvite. EDX was also completed for the crystals produced by *B. pumilus* (**a**), *H. salinarum* (**b**) and present overlapping results to figure (**d**).

Regarding solid precipitate formation, further analysis using SEM and EDX allowed the identification of crystals as struvite in tests between pH 7.3 to 8.3 (Fig. 3). The bio-struvite produced displayed a orthorhombic shape typical of struvite crystals^4^, with sizes between 0.10–0.40 mm (Fig. 3). Some of the crystals seemed to result from the aggregation of two crystals resulting in an X-shaped crystal with sizes on the higher values of the observed range of sizes, and also rhombohedral crystals with sizes in the lower values. Both these forms have been described as typical crystal habits of struvite^32^. Bio-struvite production was measured taking in consideration the values measured in the un-inoculated control and the maximum values reached of: 112 mg/L for *B. pumilus* in the test with initial pH of 7.8, 107 mg/L for *H. salinarum* in the test with initial pH of 7.3 and 77 mg/L for *B. antiquum* in the test with initial pH of 7.3.

**Figure 4:**
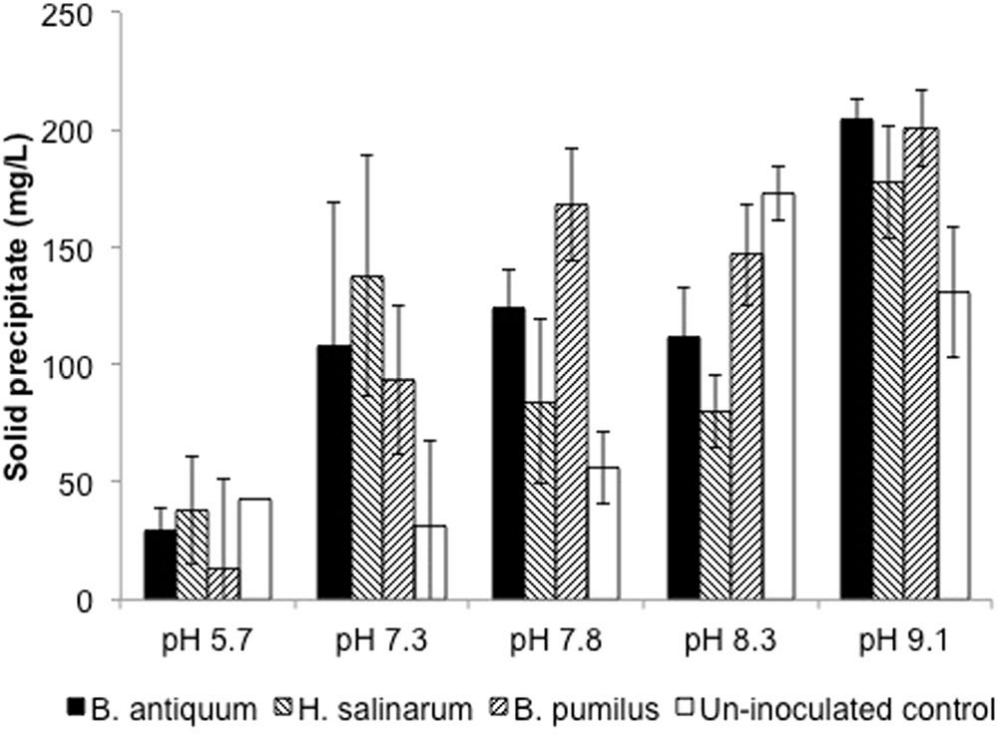
Solid precipitate collected after 6 days of incubation of pure cultures of the selected bacteria in magnesium supplemented sludge dewatering liquors at different initial pH values. Error bars represent standard deviation obtained from triplicate tests.

At pH of 5.7 no statistical differences were identified between the inoculated group and the control tests. At pHs above 8.3 the solid precipitate formation was higher in the controls, reaching a maximum of 173 mg/L (Fig. 4). pH values above 8.5 are known to be optimal for chemical struvite precipitation^4,33,34^, hence, it was expected that solid precipitate formation was high in the controls due to the formation of precipitates through chemical routes. At pHs above 8.3, chemical precipitation becomes significant and it is not possible to differentiate between chemical and biological struvite production and hence biological struvite precipitation is not relevant. Nevertheless, the typical pH of sludge dewatering liquors is <8.3 and under these conditions it is possible to recover struvite using biomineralisation^25^.

At pH 9.1, an increased formation of solid precipitate was observed in the inoculated tests relative to the controls, with the highest difference observed for *B. antiquum*, with 74.2 mg/L above the control. The precipitates recovered in the control had an amorphous nature and the precipitates formed were identified as amorphous calcium compounds, by morphological analysis^23^. Nevertheless, the precipitates recovered from the inoculated tests, showed a mix of amorphous and crystalline structures indicating the influence of bacteria at a high of pH > 9.1. This constitutes another significant role microorganisms in biomineralisation, their ability to act as heterogeneous nucleation sites^35^, enhancing the production of precipitates.

### Bio-struvite production rate

Bio-struvite formation over a 10-day period was assessed using sacrificial bottles in order to understand the production rate over time. In these tests, the pH was controlled either through pH correction with chemicals (Experiment A, pH of 7.9), or by limiting CO_2_ degassing in closed serum bottles (Experiment B, pH of 7.8). Both strategies were tested with the aim of reducing chemical changes in the composition of the sludge dewatering liquors. In both tests, the headspace was 75 mL allowing for dissolved oxygen concentrations being always above 5 mg/L in both Experiments A and B. As a result, no oxygen depletion was observed in any of the tests.

**Figure 5:**
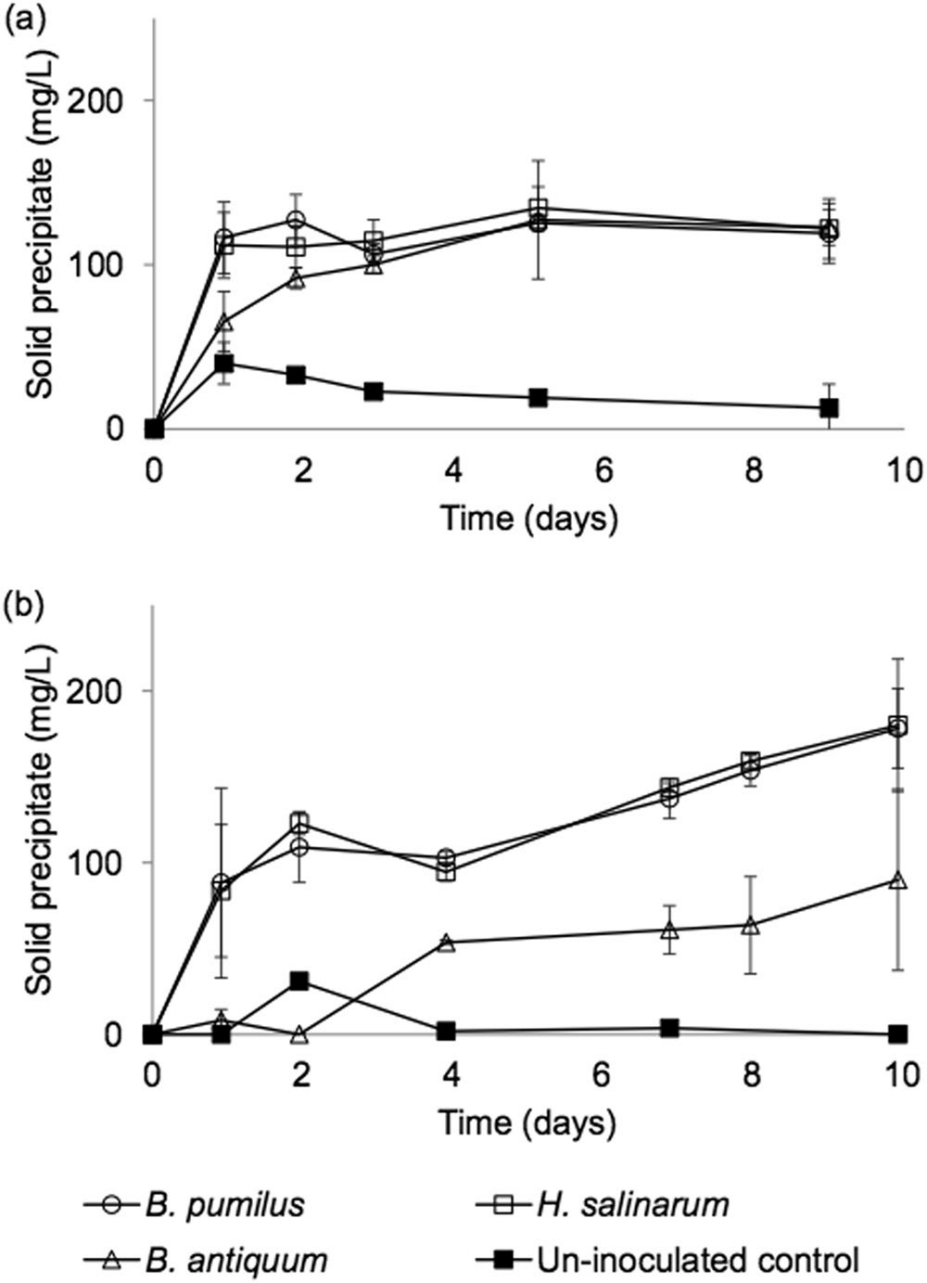
Solid precipitate production when incubating the selected bacteria in: (**a**) pH controlled through acid addition (Experiment A) and (**b**) pH controlled by limiting degassing in closed serum bottles (Experiment B). Controls were not inoculated with bacteria. Error bars represent standard deviation obtained from triplicate tests.

Inoculated and control tests presented clearly different results as limited amounts of solid precipitates were recovered in the non-inoculated controls (Fig. 5). In the inoculated bottles, the precipitates produced were analysed with SEM and EDX which allowed the identification of crystals as bio-struvite with the same characteristics as the crystals presented in Fig. 3. The concentrations of PO_4_, NH_4_ and Mg^2+^ were measured at the end of the 10 days incubation in both Experiments A and B (Table 3). Considering the struvite stoichiometric ratio of Mg:NH_4_-N:PO_4_-P; 1:1:1, the content of PO_4_ recovered as bio-struvite in Experiment A represented 92, 102, and 94% of the initial PO_4_. However, the removal of PO_4_ was only 51, 47, and 52% of the initial PO_4_ measured at 39.1 mg P /L. In Experiment B, the content of PO_4_ in the recovered bio-struvite represented 69, 71, and 35% of the initial PO_4_ (35.5 mg P /L) for *B. pumilus*, *H. salinarum*, and *B. antiquum*, respectively, whilst the overall removal of PO_4_ from the sludge dewatering liquors was only 16, 16, and 23% of the PO_4_ available initially. Final PO_4_ concentrations for Experiment A were 19.1, 20.7, and 18.9 mg P /L, for *B. pumilus*, *H. salinarum*, and *B. antiquum*, respectively, and in Experiment B they were 20.8, 21.0, and 24.0 mg P /L, for *B. pumilus*, *H. salinarum*, and *B. antiquum*, respectively (Table 3). These final concentrations of PO_4_ were one order of magnitude higher than the final concentrations observed in the first report of bio-struvite formation, where *B. pumilus* and *B. antiquum* lowered the PO_4_ concentration to 2.1, and 1.5 mg P /L, respectively^36^. These results suggest that the bacteria were able to use other forms of P present in the sludge dewatering liquors, such as organic P and polyphosphates. Others have reported that during anaerobic digestion of BNR biomass, polyphosphate or its hydrolysed forms are released into the liquors, thereby contributing to the phosphorus total concentration^37^. Further work is necessary to understand the types of P used by the bio-struvite producing bacteria and their favoured sources in order to predict the potential for bio-struvite production in WWTP. Another challenge is to identify the optimal conditions and factors that can enhance the growth of the bio-struvite producing bacteria in the sludge dewatering liquors mixed cultures and thus increase the potential for application of this process at full-scale.

**Table 3:** Initial and final concentrations of PO_4_, NH_4_, and Mg in sludge dewatering liquors before and after 10 days of incubation with selected bacteria.

		PO_4_ (mg P/L)	NH_4_ (mg N/L)	Mg (mg/L)
Experiment A				
Initial		39.1 ± 0.5	708 ± 6	51.7 ± 0.8
Final	*B. antiquum*	18.9 ± 1.3	711 ± 11	34.1 ± 1.3
	*B. pumilus*	19.1 ± 0.2	704 ± 3	35.8 ± 1.6
	*H. salinarum*	20.7 ± 0.4	711 ± 3	36.4 ± 1.0
Experiment B				
Initial		35.5 ± 1.0	887 ± 42	47.5 ± 0.1
Final	*B. antiquum*	24.0 ± 2.8	827 ± 16	30.7 ± 1.2
	*B. pumilus*	20.8 ± 1.1	814 ± 6	28.8 ± 0.5
	*H. salinarum*	21.0 ± 2.8	814 ± 20	29.2 ± 1.0

In Experiment A the pH was controlled through acid addition and in Experiment B the pH controlled by limiting degassing in closed serum bottles.

In Experiment A, the bio-struvite production was similar for all inoculated tests reaching 131 ± 20, 134 ± 20, and 135 ± 12 mg/L for *B. pumilus*, *H. salinarum*, and *B. antiquum*, respectively (Fig. 5a). In Experiment B, using closed serum bottles, bio-struvite production was similar for *B. pumilus* and *H. salinarum*, reaching 196 ± 25 and 198 ± 42 mg/L, respectively. *B. antiquum* demonstrated a lower production of bio-struvite, 99 ± 58 mg/L, although it is not clear why it was lower than in experiment A.

Maximum productivities in Experiment A were observed after the first day for all bacteria: 138, 132, and 77 mg/L.d, for *B. pumilus*, *H. salinarum*, and *B. antiquum*, respectively. In Experiment B, *B. pumilus* and *H. salinarum* reached 106, and 100 mg/L.d within 1 day, and *B. antiquum* required 4 days to reach a maximum productivity of 15 mg/L.d. Overall, the results indicated that 75% of the bio-struvite production took place in the first 2 days of incubation in Experiment A. This is an important result to note when considering scaling up this process, as long retention times imply larger tanks and increased costs. A retention time of 1–2 days is long in comparison to mainstream wastewater treatment processes such as activated sludge or membrane bioreactors, that allow for treatment of the wastewater in the order of hours^38^. Retention times for chemical struvite precipitation in fluidised reactors range from 0.5–9 h^4,22^. Nevertheless, processes that deal with low volumes can be economically feasible, such as anaerobic digestion (retention time between 10–15 days) and other processes that deal with the treatment of sludge dewatering liquors such as anammox processes with retention times of up to 20 days^7^. The findings from this study complement existing literature^10^ on the possible application of the bio-struvite process to wastewater sources including: low-P sludge dewatering liquors not viable for use with chemical struvite precipitation; wastewater streams not typically used for recovery (e.g.: settled wastewater) and streams with pH < 8.3; no need to add chemicals, large struvite crystals are produced (0.1–0.4 mm). The estimation of the capital and operational costs for bio-struvite recovery needs to be estimated once the process is developed further and more information is available on suitable reactor designs, operation conditions, etc. Ideally, bio-struvite recovery would take place in in mixed culture reactors enriched with biological struvite producing bacteria. Bacteria enrichment is accomplished by a combination of specific bioreactor design and operational conditions that results in the selective pressure that favours the growth rate of the target microorganisms. If enrichment were not achieved, then the production of bio-struvite would need to take place in sterilized sludge dewatering liquors. The latter is currently practiced in anaerobic digester with thermal hydrolysis (e.g.: the CAMBI process is used for the thermal hydrolysis of non-digested wastewater sludge. The sludge is subjected to 165–170 °C for 20 minutes at 90–100 PSI^39^ (which is sufficient for sterilisation). The target costs for the biological struvite recovery should be similar or lower to the chemical struvite recovery that has been estimated at 2.0 EUR2016/(person.yr) with sludge liquors with >100 mg P/L, up to 4.0 EUR2016/(person.yr) with liquors <100 mg P/L^40^.

## Conclusions

Sludge dewatering liquors can be used to produce bio-struvite, however, pH can impact the growth of the selected bacteria. The bacteria investigated were able to grow in sludge dewatering liquors at a wide range of pHs from 5.7 to 9.1. Bio-struvite production was observed at pHs between 7.3 to 7.8 with productions between 135–198 mg/L above non-inoculated controls. At pHs above 8.3, chemical precipitation becomes significant and it is not possible to differentiate between chemical and biological struvite production. Nevertheless, the typical pH of sludge dewatering liquors is <8.3 and under these conditions it is possible to recover struvite using biomineralisation. Results indicated that bio-struvite production processes are likely to be applicable in WWTP with sludge dewatering liquors with a neutral pH and low dissolved CO_2_ concentrations.

## Electronic supplementary material

Supplementary Information
