# Supplementary material for: The role of pH on the biological struvite production in digested sludge dewatering liquors
Source: Sci Rep. 2018 May 8;8:7225. doi: 10.1038/s41598-018-25431-7 (PMC5940904; doi:10.1038/s41598-018-25431-7)
Supplement: Supplementary file 1 — Supplementary Information [file 41598_2018_25431_MOESM1_ESM.doc]

**Supplemental information:**

# The role of pH on the biological struvite production in digested sludge dewatering liquors

Francisco Simoes^a^, Peter Vale^b^, Tom Stephenson^a^, Ana Soaresa[[1]](#footnote-2)

^a^Cranfield Water Science Institute, Cranfield University, Cranfield, MK43 0AL, UK

^b^Severn Trent Water Limited, Severn Trent Centre, 2 St John's Street, Coventry, CV1 2LZ, UK

Corresponding author: Ana Soares, a.soares@cranfield.ac.uk.

**Supplemental information:**

# The role of pH on the biological struvite production in digested sludge dewatering liquors

In order to assess if high dissolved CO_2_ concentrations were responsible for changes in pH, tests were completed to measure the dissolved CO_2_ in the studied sludge dewatering liquors.

# Materials and methods

## Degassing of CO_2_ from sludge dewatering liquors

The amount of CO_2_ available in the sludge dewatering liquors to be released as a gas was estimated according to the methodology described by Hafner and Bisogni (2007)^1^. Twenty millilitres of sludge dewatering liquors were placed in serum bottles and hydrochloric acid 2 M was added at 4 different levels: no acid, 500 μL, 700 μL, and 1000 μL. After acid addition the sludge dewatering liquors were autoclaved in closed and open serum bottles and these were compared with serum bottles kept static outside the autoclave. After 4 hours, the pH and the CO_2_ fraction of the headspace of closed serum bottles were measured in a CSi 200 series gas chromatograph setup as described by McLeod et al. (2013)^2^. All tests were completed in triplicate.

## Release of CO_2_ during post anaerobic digestion treatment

To assess the impact of post anaerobic digestion treatment on the amount of CO_2_ release from the digestate and corresponding changes in pH, two column tests were setup to simulate the conditions in aerated and non-aerated storage. Digestate was collected from three anaerobic digesters treating primary and BNR sludge and mixed in equal proportions. The digestate was placed in two open Perspex plastic cylindrical columns (0.96 m x 0.10 m). One of the columns was left standing for 22 days to simulate the post anaerobic digestion storage conditions of the full-scale WwTW sampled. In the second column the digestate was aerated at 0.3 L/min compressed air for 20 days simulating aerated post anaerobic digestion storage conditions predominant in other WwTWs. Digestate samples were collected after 11, 20 and 22 days for the non-aerated column and after 2, 11 and 20 days for the aerated column. Twenty millilitres of sample were placed in serum bottles, the pH and the dissolved CO_2_ were also measured.

Sterilization of the sludge dewatering liquors the pH increased from 7.8 to 9.8 (Table S1). Filter sterilisation was also tested and although this method led to a more moderate pH change (from 7.8 to 8.5), after inoculation with the selected bacteria and incubation by agitation, the pH raised to 9.4 ± 0.1.

# Results

Sterilization of the sludge dewatering liquors the pH increased from 7.8 to 9.8 (Table S1). Filter sterilisation was also tested and although this method led to a more moderate pH change (from 7.8 to 8.5).

Table S1. Characteristics of the sludge dewatering liquors collected from a full scale site before and after sterilization treatments.

|  | pH | COD (mg/L) | NH_4_ (mg N/L) | PO_4_ (mg P/L) | Mg^2+^ (mg/L) |
| --- | --- | --- | --- | --- | --- |
| Sludge dewatering liquors collected from full-scale site | | | | | |
|  | 7.8 | 455 ± 3 | 825 ± 66 | 44.5 ± 2 | 15.2 |
| Sludge dewatering liquors after processing | | | | | |
| Centrifugation | 8.3 | 327 ± 4 | 799 ± 14 | 27 ± 1 | 9.6 |
| Sterilised by autoclave | 9.7 | 449 ± 11 | 516 ± 6 | 58 ± 0 | – |
| Sterilised by filtration | 8.5 | – | 736 ± 11 | 23 ± 1 | 2.1 |

After 22 days the digestate kept in anaerobic conditions had a pH of 8.3 and released enough CO_2_ to enrich the headspace to 4.7% CO_2_, whilst the digestate from the aerated column released 4.1% CO_2_ with a pH of 8.7 from day 2 onwards (Figure S1). Results show that the potential for CO_2_ release was higher in the un-aerated test (Figure S1).

These results indicate that post anaerobic digestion treatment is a relevant factor for the implementation of a bio-struvite process and pH of the sludge dewatering liquors.


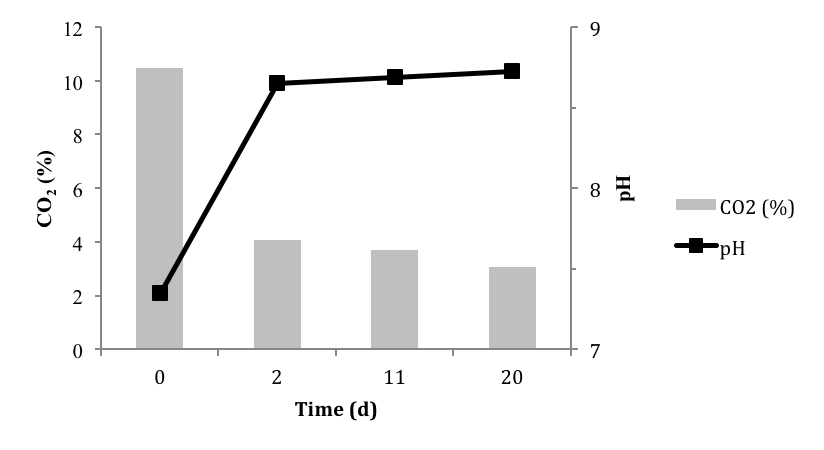


a)

b)


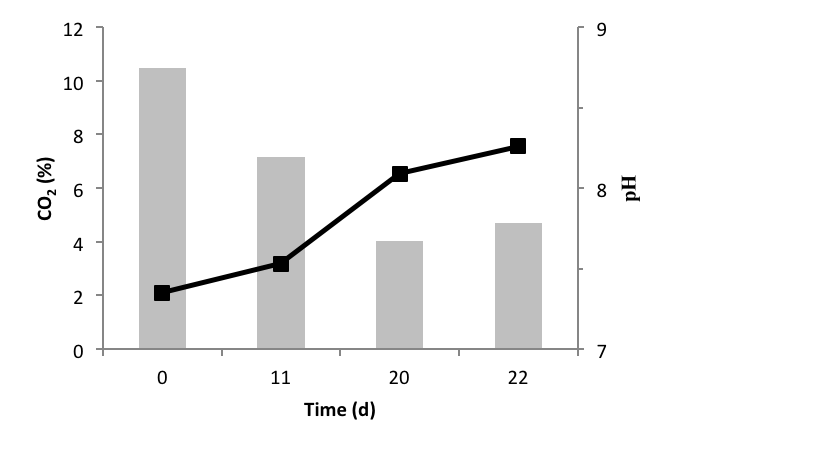


Figure S1Aanaerobic digestate pH and released CO_2_ fraction for two distinct post anaerobic-digestion treatments: a) aerated; b) non-aerated.

# References

1.Hafner, S. D. & Bisogni, J. J. A Simple Method for Measurement of Inorganic Carbon Concentration and Carbonate System Alkalinity in Anaerobic Digesters. *Agric. Eng. Int. CIGR Ejournal. Manuscr. EE 06 008* **IX,** 1–15 (2007).

2. McLeod, A., Jefferson, B. & McAdam, E. J. Quantifying the loss of methane through secondary gas mass transport (or ‘slip’) from a micro-porous membrane contactor applied to biogas upgrading. *Water Res.* **47,** 3688–3695 (2013).

1. Corresponding author at Cranfield Water Science Institute, Cranfield University, Vincent Building, Cranfield, Bedfordshire, MK43 0AL, UK. Tel.: +44 (0) 1234 758121. E-mail address: a.soares@cranfield.ac.uk (A. Soares). [↑](#footnote-ref-2)
